# Retraction: Lack of emotional experience, resistance to innovation, and dissatisfied musicians influence on music unattractive education

**DOI:** 10.3389/fpsyg.2026.1863494

**Published:** 2026-05-11

**Authors:** Frontiers Editorial Office

Following publication, concerns were raised regarding the validity of the data in the article. The authors failed to provide the raw data or a satisfactory explanation during the investigation, which was conducted in accordance with Frontiers' policies. Given the concerns, and the lack of raw data, the editors no longer have confidence in the findings presented in the article. This retraction was approved by the Chief Editors of Psychology and the Chief Executive Editor of Frontiers. The authors do not agree to this retraction. Frontiers would like to thank the concerned reader who contacted us regarding the published article.

